# Supplementary figures and images for: Metabolic Profiling Reveals That the Olfactory Cues in the Duck Uropygial Gland Potentially Act as Sex Pheromones
Source: Animals (Basel). 2022 Feb 9;12(4):413. doi: 10.3390/ani12040413 (PMC8868514; doi:10.3390/ani12040413)

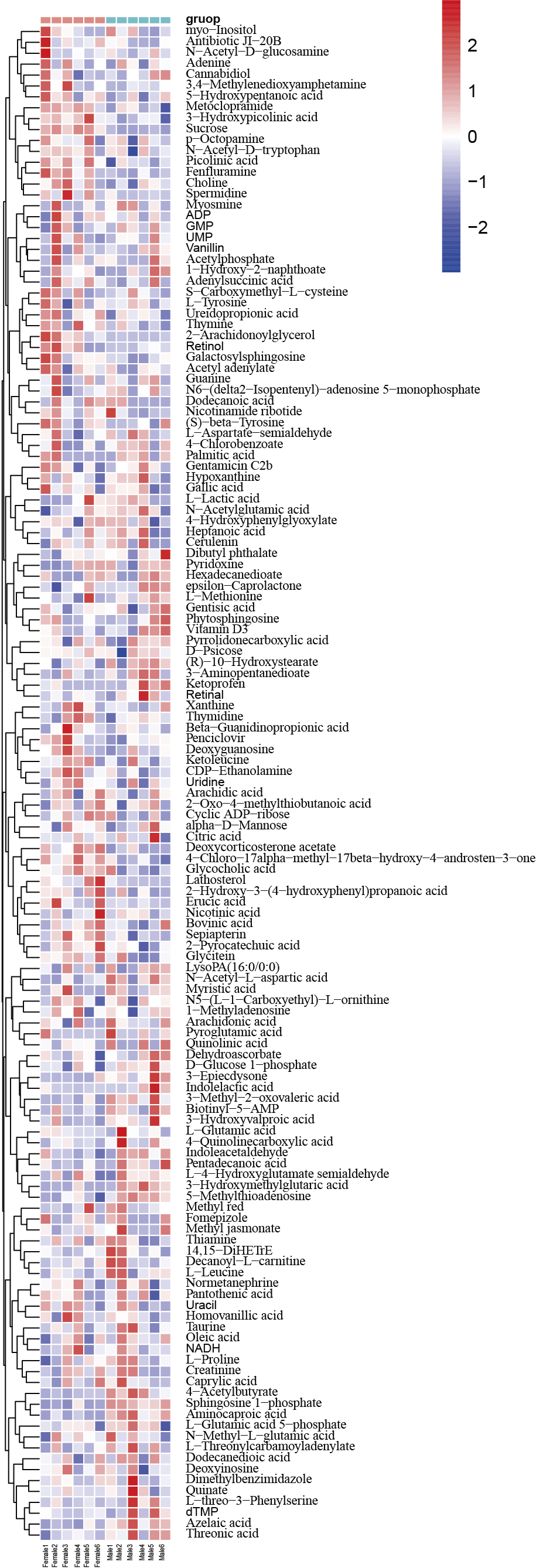

Supplement: Supplementary file 1 [file animals-12-00413-s001.zip › animals-1516369-Supplementary/Supplementary Figure S1.tif]
